# Peritonitis following unsafe abortion: a retrospective study in a tertiary health facility in North Central Nigeria

**DOI:** 10.11604/pamj.2020.37.354.22775

**Published:** 2020-12-18

**Authors:** Adedire Timilehin Adenuga, Oluwatosin Wuraola Akande

**Affiliations:** 1 Department of Surgery, University of Ilorin Teaching Hospital, Ilorin, Nigeria,; 2 Department of Community Medicine, University of Ilorin Teaching Hospital, Ilorin, Nigeria

**Keywords:** Unsafe abortion, bowel injury, peritonitis, laparotomy, stoma, Nigeria

## Abstract

**Introduction:**

surgical complications following unsafe abortion (UA) are not uncommon and are associated with high morbidity and mortality in developing countries. The commonest need for the general surgeon following UA is after a diagnosis of peritonitis which can occur following use of sharp objects introduced through the vagina. This study aims to highlight the presentation, management types and outcome of patients who presented with peritonitis following UA.

**Methods:**

this study is a retrospective review of cases of peritonitis following UA seen over 4 years from January 2015 to December 2019 in a tertiary health facility in North Central Nigeria.

**Results:**

a total of 14 patients with peritonitis following UA were included in the study. The mean age of patients who presented was 27.4 years (19-40 years) with a mean estimated gestational age at abortion of 7.8 weeks. The average time from the UA procedure till presentation at the hospital was 8.6 days. There were 9 bowel injuries and 5 pelvic abscesses. A total of 3/9 patients had primary resection and anastomosis while 6/9 had stoma formed as part of their management. Pelvic abscesses were drained. In patients with bowel injury, those who had primary anastomosis had a 100% incidence of enterocutaneous fistula formation with associated sepsis requiring repeat exploration and formation of stoma. Mortality in this group was 67% (2/3) compared to the 0% (0/6) mortality rate seen in patients who had stoma. The overall mortality was four out of fourteen patients (28.6%).

**Conclusion:**

peritonitis following UA is associated with marked morbidity and mortality as many of the patients present late. Initial preoperative resuscitation and stabilization should be followed by a swift laparotomy. Patients with bowel injury who had primary anastomosis had higher morbidity, reoperation rates and mortality than patients who had stomas.

## Introduction

Abortion is the discontinuation of pregnancy, either spontaneously or by intervention before the foetus reaches viability [1]. In Nigeria, abortion is illegal except it is done to save the life of the mother [2]. The abortion laws preclude induced (criminal) abortion with punishment for both the woman and the personnel performing the abortion procedure. Those who violate the law face a steep penalty, if caught, the patient and the performer risk a seven and fourteen year jail term respectively [2,3]. There is still a high unmet need for contraceptives in Nigeria and as such, patients with unwanted and unplanned pregnancies seek abortions may from questionable (health) personnel [4,5]. Unsafe abortions (UAs) are defined by the World Health Organisation as those performed either by individuals lacking the necessary skills or in an environment that does not conform to minimal medical standards, or both [6]. Many of the unsafe abortions done in our environment include the use of all kinds of sharp instruments that are passed through the vagina. These instruments may perforate the uterus and extend into the peritoneal cavity. Complications from unsafe abortion may be associated with injuries to other intraabdominal viscera which may require the intervention of the general surgeon. The spectrum of general surgical considerations may range from pelvic collections to gut perforation with peritonitis. Other general surgical presentations include: mesenteric vascular injury with extensive devascularisation of large segments of the bowel and small bowel evisceration/exteriorization.

The widespread poor financial outlook and lack of basic health insurance may mean that these patients present days after the botched procedure, usually as a means of last resort with poor physiologic states, resulting abysmal prognosis [7]. Complications of unsafe abortion are particularly unsavoury because majority of these females come from a place of shame and ostracization to have these clandestine procedures done by unskilled workers. These unskilled personnel use unsanitary sharp instruments, exposing patients to the highest form of injury and trauma. There is no consensus on the particular procedure to be done in the setting of bowel injury following UA. The options of simple repair, resection and anastomosis and exteriorization largely depend on the surgeon´s preference and hemodynamic stability of the patient [8]. The choice of procedure done at laparotomy for complications of unsafe abortion go a long way in determining morbidity or mortality. This paper uses the data from patients who were managed over three years in a tertiary teaching hospital in North Central Nigeria for complications of UA to highlight the presentation, management types and outcome of patients.

## Methods

**Setting:** the study was conducted at the Division of General Surgery, University of Ilorin Teaching Hospital (UITH), Ilorin, Kwara State of Nigeria. It is a tertiary centre that receives patients from Kwara State and neighbouring States of Kogi, Oyo, Osun and Niger States. The division is responsible for providing general surgical care to patients who present on their own or are referred from other health facilities.

**Study design and patient selection:** this is a descriptive retrospective study that consisted of fourteen patients who were co-managed by both the gynaecology and general surgery teams of the University of Ilorin Teaching Hospital for complications following UA.

**Data collection procedure and analysis:** this study included patients who presented with complications of UA between January 2015 and December 2019. A total number of 14 patients met the selection criteria and their records were retrieved and relevant data extracted. Following proper clinical evaluation, preoperative resuscitation and relevant investigations, all patients had emergency exploratory laparotomy via a midline incision. Data of interest included age, estimated gestational age at abortion, duration in days from abortion till presentation, duration of hospital stay, location and type of injury, type of procedure done, perioperative blood requirement, intensive care unit admission, post operative morbidity and mortality.

## Results

**Clinical presentation:** the mean age of the patients was 27.4 ± 6.2 years (Range = 19 - 40). The mean Estimated Gestational Age (EGA) at abortion was 7.8 weeks (Range = 5 - 11). The duration in days from the botched procedure to presentation at our facility was an average of 8.6 days (Range = 1 - 21). All the patients presented with features of generalized or localized peritonitis and were resuscitated with intravenous fluids, patient had pre and post-operative intravenous antibiotics with ciprofloxacin and metronidazole, parenteral analgesics, urethral catheterization ensuring adequate urine output. All patients had a plain chest radiograph and abdominal ultrasound done before laparotomy.

**Figure 1:**
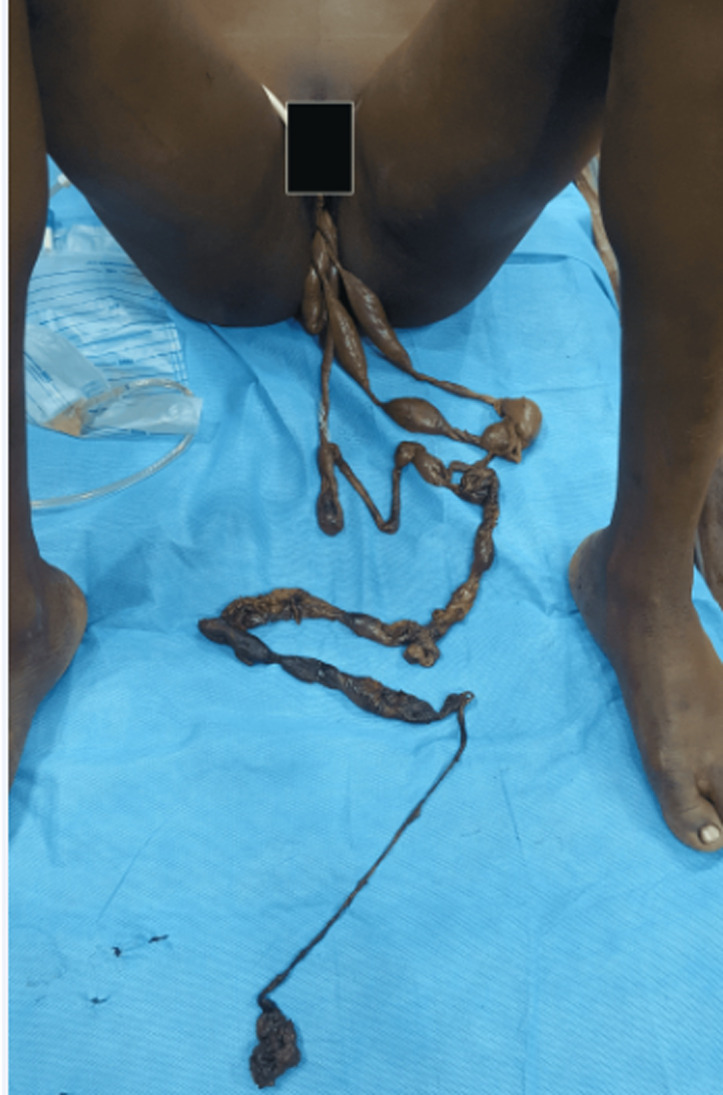
showing vaginal evisceration following unsafe abortion

**Operative details:** ileal injury was the commonest presentation, accounting for 43% (6/14) of all cases. Patients with pelvic abscesses accounted for 36% (5/14) of all cases while patients with sigmoid injury, rectal injury and vaginal evisceration each accounted for 7% (1/14 each) (Table 1). One patient presented 36 hours after UA with vaginal evisceration and about 100cm of ileum pulled from the vagina when a health attendant mistook it for foetal cord (Figure 1). Five patients had pelvic abscesses requiring drainage without any associated bowel injury. Other procedures done are as listed in Table 2 below. The uterine perforation was located at the posterior and fundal aspects in 57.1% (8/14) and 42.9% (6/14) of cases respectively. An improvised drain using size 20FR urethral catheter was placed in the pouch of Douglas after copious peritoneal lavage and brought out via a separate stab wound. Povidone Iodine soaked wound dressings were changed depending on degree of post-operative wound discharge. Three out of the 14 patients required post-operative ICU care due to poor post-operative state. A total of 10 of the 14 patients required perioperative blood transfusion with patients receiving an average of 3 pints of blood (range = 2 - 6).

**Table 1:** location of injury following unsafe abortion

Site of injury	Number	Percentage
Ileum	6	43%
Pelvic abscess	5	36%
Sigmoid	1	7%
Rectum	1	7%
Vaginal evisceration	1	7%
**Total**	14	100%

**Table 2:** different procedures done for peritonitis following unsafe abortion

Procedure	Number	Percentage
Ileostomy	4	29%
Drainage of pelvic abscess	5	36%
Resection and anastomosis	3	21%
Colostomy	2	14%
**Total**	14	100%

**Figure 2:**
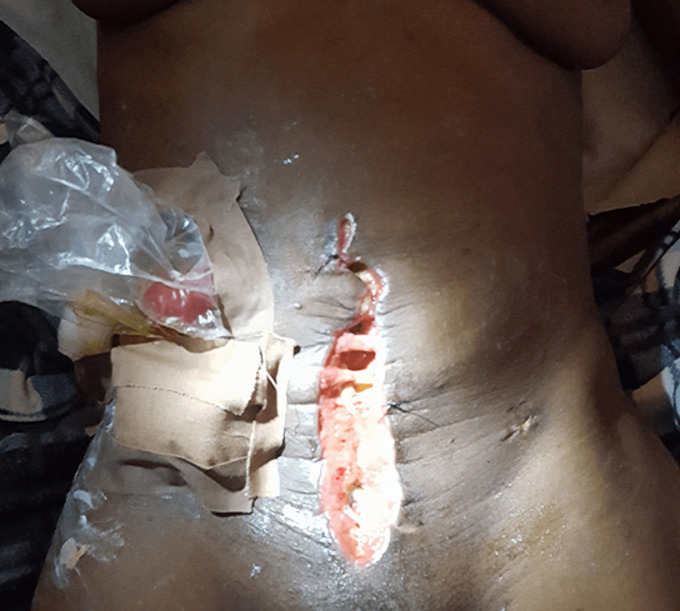
burst abdomen following laparotomy

**Outcomes:** the mean number of days spent on admission was 28 days (range 10 - 48). All patients except one had varying forms of surgical site infection (93%). Figure 2 shows wound dehiscence following laparotomy for UA. Other morbidities are as listed in Table 3. There were four mortalities accounting for 28.6% of all the cases in this series. All mortalities were due to sepsis. A total of 3 patients had primary resection and anastomosis and all (100%) developed enterocutaneous fistulas requiring reoperation and creation of stomas. The mortality in this group of patients was 67% (2/3). The characteristics of patients who required reoperation are as listed in Table 4.

**Table 3:** post-operative morbidity

Morbidity	Number of cases
Intra-abdominal collection	4
Enter cutaneous fistula	3
Burst abdomen	2
Post-operative pneumonia	1
Short bowel syndrome	1

**Table 4:** characteristics of re-operated patients

	Initial diagnosis	Fecal peritonitis at initial surgery	Initial Procedure done	Complication	Procedure at reoperation	Days on admission	Mortality
J.L	Multiple ileal perforations	Yes	Ileal Segmental resection and anastomosis	ECF	Divided ileostomy	42	Yes
O.B	Extensive ileal injury	Yes	Ileal segmental resection and anastomosis	ECF	Divided ileostomy	38	Yes
A.F	Multiple ileal perforations	Yes	Ileal segmental resection and anastomosis	ECF	Divided ileostomy	40	No
S.H	Pelvic abscess	No	Drainage and peritoneal lavage	Intra-abdominal abscess	Drainage and peritoneal lavage	12	Yes

**Follow up:** seven patients had reversal of their temporary stoma and they were followed up for an average of 3 months (average 1-5). There were no complications recorded in the time period.

## Discussion

Peritonitis from unsafe abortion, though rare in the developed world is not uncommon in this setting. The current restrictive abortion laws present in Nigeria exposes patients to having the procedure done by unskilled workers usually followed by its attendant morbidity and mortality. Some of the unsafe methods used to terminate a pregnancy include the use of foreign bodies in the uterus such as twigs, cloth hangers, chicken bones, cassava stalk, and herbal preparations [9,10]. Perforation of the uterus and entry into the peritoneal cavity with these heavily contaminated devices/agents kickstarts a long and turbulent septic illness which may require the intervention of the general surgeon. The mean age of patients in this study was 27.8 years which is similar to the mean age of 25.5 years in Malawi patients with induced abortions [11]. In this study, the patients spent an average of 8 days after the abortion procedure before presentation at the hospital. A study in Tanzania reported 6 days between abortion and presentation at a health facility [12]. Some of the reasons which may be adduced to this include: failure of recognition of the seriousness of the diagnosis, sociocultural factors as well as financial limitations. This lag between the botched procedure resulting in injury and presentation means that they arrive the emergency room in poor preoperative physiologic states (such as anemia and sepsis) which may be partly responsible for the poor outcomes seen. Furthermore, in this study the mean length of hospital stay was 28 days. Morbidities such as wound infection, burst abdomen and reoperations mean that these patients are likely to have four times the duration of hospital stay than that of patients with laparotomy wound complications for other conventional cases such as ruptured appendicitis; this is in addition to an increase in hospital costs and risk of nosocomial infections [13]. All patients in this study had their UA done during the first trimester with a mean EGA of 7.8 weeks. This is congruent with studies that have shown that the commonest period abortion is carried out is in the first trimester. Conversely, this is in contrast to the study done in Tanzania where over 70% of patients terminated their pregnancy in the second trimester [12]. Worse outcomes have been reported when the abortion is done during the second and third trimester [14,15]. The reasons cited for delay in pregnancy termination include ignorance, tardiness in decision making as well as the highly restrictive laws and secrecy associated with abortion in the country.

In this study, the ileum was the commonest site of injury in 43% (6/14) of cases, the sigmoid colon and rectum were perforated in two patients. The relative mobility of the small bowel within the peritoneal cavity makes it susceptible to being injured while relatively fixed structures such as the rectum may be perforated as part of posterior uterine perforation. This finding is similar to the report by Mabula *et al*. where bowel perforations (ileal, sigmoid, rectum) occurred in 57% (8/14) of patients [12]. In a study by Megafu *et al*. 73.3% of patients with peritonitis requiring laparotomy following abortion had bowel perforation. Furthermore, 57.1% of all uterine perforations were located posteriorly and may explain the prevalence of posteriorly located injury and pathology seen - sigmoid, rectal as well as pelvic abscesses at the pouch of Douglas [16]. This is similar to the work by Jhobta *et al*. where they recorded posterior perforation in 63.6% (7/11) of patients who had UA [17]. These findings suggest that the general surgeon needs to properly evaluate and be thorough in assessing the uterus for perforations. All but one patient in this series (93%) developed postoperative surgical site infection (SSI) which is not unexpected giving that all patients underwent emergency procedures with creation of dirty wounds on a background of sepsis, some with fecal peritoneal contamination containing virulent gut organisms. There is a higher risk of SSI following surgery on patients presenting with preoperative shock, sepsis, anaemia, and long-standing illnesses [18]. Many of these factors were seen in these patients. Superficial SSI was managed with regular wound dressings, two patients had burst abdomen which were repaired. SSI is a major morbidity leading to prolonged hospital stay, increased hospital costs in the short term, with incisional hernia, infertility, chronic post-operative pain and adhesive intestinal obstruction being long term complications [19].

Almost three quarter (71.4%) of the patients in this study required perioperative blood transfusion. In addition to the preoperative delay and bleeding associated with UA, some of these patients might have had some form of nutritional malnutrition with suboptimal blood reserves which became severely depleted with the onset of the injury. The choice of surgery done on a patient with unsafe abortion goes to a long extent in determining outcome as grave morbidity and mortality are associated with wrong and inappropriate surgeries [20]. In the presence of extensive/multiple bowel injuries, the intraoperative options include simple repair, resection and anastomosis, exteriorization, or repair and a proximal diverting stoma [8]. Despite its retrospective nature, results from this study show that diversion in form of stoma creation as the procedure of choice. Patients who had resection and primary anastomoses had a 100% rate of enterocutaneous fistula and all were re-operated. The mortality after reoperation was 67%. In contrast, there was no mortality among patients who had laparotomy and stoma creation. There is no consensus on the optimal management of these patients. The decision to elect for one of the above repairs depends largely on the surgeon´s experience, the hemodynamic stability of the patient and the degree of intraperitoneal soilage. Many of these patients present late, septic and anaemic with poor preoperative physiologic parameters. Intraoperatively, there may be massive purulent or gross fecal peritoneal soilage and intraoperative hypotension. Some of these patients may require post-operative Intensive care unit monitoring. All the above factors, especially for patients in our setting, require a holistic consideration that generally does not favour a primary resection and anastomosis.

In extensive ileal injuries, the choice of operative procedure, primary anastomosis or stoma creation, depends on these factors. In the setting of extensive fecal peritoneal contamination, which is seen commonly due to late presentation, resection and primary anastomosis may be precarious. A swift laparotomy with exteriorization of bowel ends may be needed despite the complications associated with the presence of a stoma. Other studies have shown that mortality is high in patients with simple repair or resection and anastomoses than patients with stomas [16,20] Diversion is preferred in left sided colonic and rectal perforation as the large bacterial load and fecal contamination make the repair precarious. The drawback of stoma placement is that, its initial management may be turbulent, and reversal requires a secondary operation which may not be easily affordable by the patient or caregivers. Copious peritoneal lavage and placement of drains are usually done. Drain use has been sine qua non in these patients due to the heavy soilage of the peritoneal cavity [10,12]. The drains were placed in the pouch of Douglas and were removed at the discretion of the operating surgeon when the output became negligible which was usually between 3-5days. The drains also served some diagnositic purpose as the presence of fecal matter in the drain was an early pointer towards an anastomotic leak. Although none of the patients in this series presented with mesenteric vascular injury, patients with mesenteric injury may require resection of large segments of bowel if there is compromise to the feeding vessel and long segment gangrene [21,22]. In a rare case, Singh *et al*. described a woman post UA who had extensive large bowel mesenteric injury which required total colectomy and terminal ileostomy [23]. The mortality rate in this study was 28.5%. All patients died from septicaemia. Similarly, a retrospective study among patients who presented with complications of unsafe abortion in a tertiary hospital in Lagos, Nigeria found that majority (80%) of mortality was due to sepsis [24].

## Conclusion

Patients presenting with features of peritonitis after unsafe abortion require aggressive preoperative resuscitation due to the poor physiologic states created by the septic process. A well-informed definitive procedure should be carried out as it has massive implications on morbidity and mortality. In this series, diversionary stoma instead of resection and anastomosis was associated with better outcomes.

### What is known about this topic

- The commonest instrument associated with peritonitis following UA are sharp and are introduced into the vagina largely by unskilled and untrained workers;
- Unsafe abortion may be complicated by peritonitis. This is particularly associated with high morbidity and mortality, as many present late and in poor physiologic states;
- The outcome of surgery following laparotomy for peritonitis following UA depends largely on the surgeon’s choice of procedure.

### What this study adds

- The demography of patients presenting with peritonitis following UA in North Central Nigeria;
- Sites of injury in peritonitis following UA;
- The different management types, operative options and outcomes.
